# A Novel Metasurface-Based Monopulse Antenna with Improved Sum and Difference Beams Radiation Performance

**DOI:** 10.3390/mi13111927

**Published:** 2022-11-08

**Authors:** Jianing Zhao, Li Hao, Hao Li, Zihao Tong, Tianming Li, Haiyang Wang, Biao Hu, Yihong Zhou, Fang Li, Cheng Fu, Qian Li

**Affiliations:** 1 College of Information Science and Engineering, Guilin University of Technology, Guilin 541006, China; 2 Guangxi Key Laboratory of Embedded Technology and Intelligent System, Guilin University of Technology, Guilin 541006, China; 3 School of Electronic Science and Engineering, University of Electronic Science and Technology of China, Chengdu 610054, China; 4 Yangtze Delta Region Institute (Huzhou), University of Electronic Science and Technology of China, Huzhou 31300, China; 5 School of Communications and Information Engineering &Artificial Intelligence, Xi’an University of Posts and Telecommunications, Xi’an 710121, China

**Keywords:** Monopulse Cassegrain antenna, metasurface, sum and difference beams

## Abstract

This paper proposes a novel metasurface-based monopulse antenna. A multimode pyramidal horn with four ports is selected as the feed of the proposed monopulse antenna. The 3-dB couplers and the optimized waveguide phase shifters are employed to design the monopulse comparator. In order to obtain good sum and difference beams performance, metasurfaces are mounted on a bowl structure to radiate the electromagnetic wave from the sub-reflector. An experimental prototype of the proposed design has been fabricated and measured at Ku-band. The measured results show that the gain ratio between the sum and difference beams is 2.8 dB and 3.7 dB, respectively. More importantly, the peak gain of the sum beam at 16 GHz is 27.1 dB, without considering the loss of the comparator, with a corresponding aperture efficiency of about 41.4%. This indicates that the proposed structure is beneficial for improving the sum and difference beams performance of the monopulse antenna, which is suitable for tracking platforms.

## 1. Introduction

Monopulse antennas with sum and difference beams (which are also denoted as Σ and Δ) can realize high direction-finding accuracy, as military tracking systems use this antenna technology extensively. The Σ configuration is usually a narrow beam with high gain and the Δ configuration generates a hollow beam with a deep null in boresight. The Σ and Δ beams provide the distance and angle of the target, respectively [1]. To reach the requirements, Cassegrain antennas or reflector antennas with four-horn feeds are typical candidates due to their simple design and good performance [2,3,4]. These antennas have curved surfaces, which make them voluminous and heavy. More importantly, ensuring the shape accuracy of these curve surfaces in practical applications is a great challenge faced by such antennas. To avoid these issues, planar monopulse antennas have been investigated. Microstrip lines and substrate-integrated waveguides (SIWs) are classic types of transmission lines, which can be used for constructing the planar monopulse applications [5,6,7,8,9,10,11,12,13,14,15]. As a result of dielectric loss and field leakage in the substrate, antennas implemented using these substrate-based transmission lines have low efficiency. The gap waveguide is made up of periodically arranged metal pins with a metal plate at a certain length affixed to them. A bandgap of a frequency range is determined by the size of the pins and the separation of the metal plate. As a result, it is possible to avoid field leakage along distribution networks as well as other undesirable effects [16]; however, due to the tiny size of the needed pins, the difficulty of producing distribution networks with these periodic pin configurations increases exponentially with size (in wavelengths) and frequency. Planar antenna arrays with radial line slots are highly efficient, mass-producible, and suitable for monopulse applications [17,18]. In most cases, this type of antenna is used to transmit circularly polarized waves.

Metasurfaces, as two-dimensional equivalents of metamaterials, can not only offer outstanding performance in sub-wavelength thickness, but also actively manipulate electromagnetic waves. Endowed with these advantages, metasurfaces have been employed in various areas. A number of studies focusing on the metasurface-based monopulse antennas were carried out [19,20,21,22,23,24,25,26]. Reconfigurable metasurfaces could realize the Σ and Δ beams consecutively [19,20,21,22,23,24], but the timeliness of the target detection is insufficient when compared to the traditional simultaneous monopulse beams. Furthermore, the aperture efficiency is relatively low. To avoid the shielding effect from the feeds, a transmissive metasurface with multi-layer microstrip patches are adopted to obtain the Σ and Δ beams [25]. The maximum gain ratio between the Σ and Δ beams is 7.56 dB, which is caused by the asymmetric Δ beams. Moreover, the measured aperture efficiency of Σ beam is 32.2%. The reflective metasurface can be also employed to construct the planar Cassegrain monopulse antenna in our previous work [26]. Although this antenna features good gain ratios between the Σ and Δ beams, the aperture efficiency of Σ beam is only 22.43%, which is mainly limited by the large aperture of the sub-reflect array and the angle-of-incidence effects. For the purpose of this overview, there is still room for improvement for the metasurface-based monopulse antennas, especially for the Σ and Δ beams radiation performance.

In this letter, a novel metasurface-based monopulse antenna is proposed. The proposed monopulse antenna adopts the Cassegrain structure and the hyperboloid is selected as the sub-reflector which avoids the effect of low sampling rate. Reflective metasurfaces with concentric square rings are mounted on a bowl structure to minimize the angle-of-incidence effects. To reduce the transmission loss, the 3-dB couplers and the optimized waveguide phase shifters have been utilized in the monopulse comparator. The metasurface-based monopulse antenna are also designed, fabricated, and measured at Ku-band. The measured results demonstrate that the proposed monopulse antenna not only has good gain ratios between the Σ and Δ beams, but also feature high-aperture efficiency.

## 2. General Structure of Metasurface-Based Monopulse Antenna

**Figure 1:**
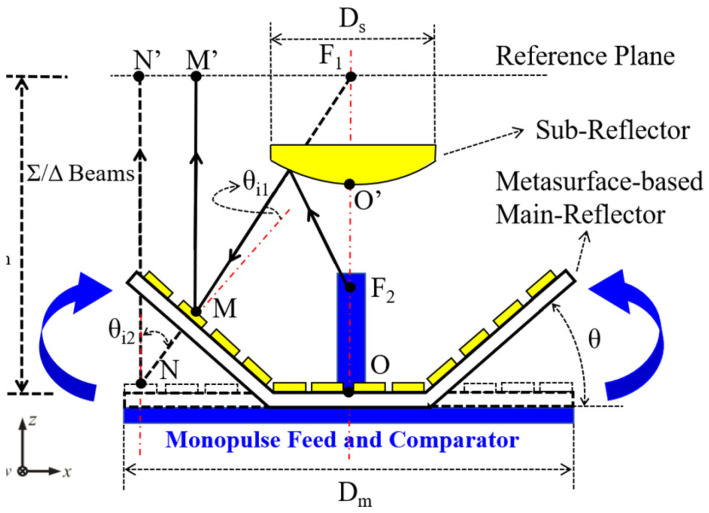
Antenna geometry schematic for the proposed metasurface-based monopulse antenna.

Figure 1 illustrates the configuration of a metasurface-based monopulse antenna. As can be seen, the proposed monopulse antenna consists of a metasurface-based main reflector, a metal hyperboloid sub-reflector, a monopulse feed of four horns, and a monopulse comparator. Main- and sub-reflector diameters are D_m_ and D_s_, respectively. The focal length is F_m_, the phase canter of the monopulse feed is located at F_2_, and the virtual focal point is located at F_1_.

As compared to our previous work [26], the following major improvements have been made: Firstly, the sub-reflector employs metal hyperboloid structure rather than metasurface. Ref. [27] points out that as the cell period decreases, the sampling becomes finer and the phase quantization becomes smaller, and so the metasurface-based sub-reflector should be designed in the same manner. In Ref. [28], the sub-wavelength cell exhibited better performance than the half-wavelength cell. Sub-reflectors are usually smaller in diameter than main-reflectors in Cassegrain antennas; therefore, the sampling-rate effects exert considerable influence on the performance of the metasurface-based sub-reflector. In order to achieve good performance, the sub-reflector adopts the metal hyperboloid structure instead of the metasurface. Secondly, for the sake of minimizing the angle-of-incidence effects, the proposed main-reflector is composed of the metasurfaces which are mounted on a bowl structure. As shown in Figure 1, compared with the planar main-reflector (the black dash line), as the folding angle θ increases, the incident angles of the cells at the edge area of the bowl main-reflector are greatly reduced, namely θ_i1_ < θ_i2_. Consequently, the bowl-shaped main-reflector could improve the radiation performance of both Σ and Δ beams in theory. Lastly, in the previous work [26], the transmission loss of the SIW-based monopulse comparator is about 3 dB, which is high transmission loss. To solve this problem, a narrow-wall waveguide monopulse comparator which concludes the 3-dB couplers and the optimized waveguide phase shifters has been utilized.

## 3. Cell Design

**Figure 2:**
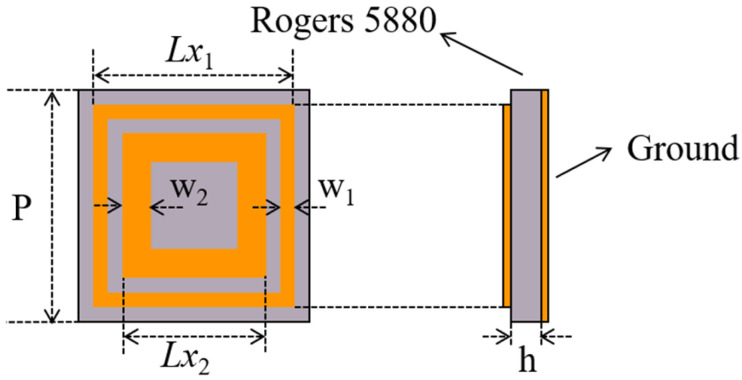
Geometry of the proposed element.

A typical concentric square-ring structure is selected as the cell of the proposed metasurface-based monopulse antenna which is shown in Figure 2. A single-layer substrate is etched with square-ring patches arranged in a square lattice with periodicity P. To achieve the low substrate loss, the substrate material adopts Rogers5880 which the thickness is h. The size of the outer square ring is *Lx*_1_, and that of the inner square ring is *Lx*_2_ = *Lx*_1_ × k. w_1_ and w_2_ represent the widths of the outer and inner rings, respectively.

**Figure 3:**
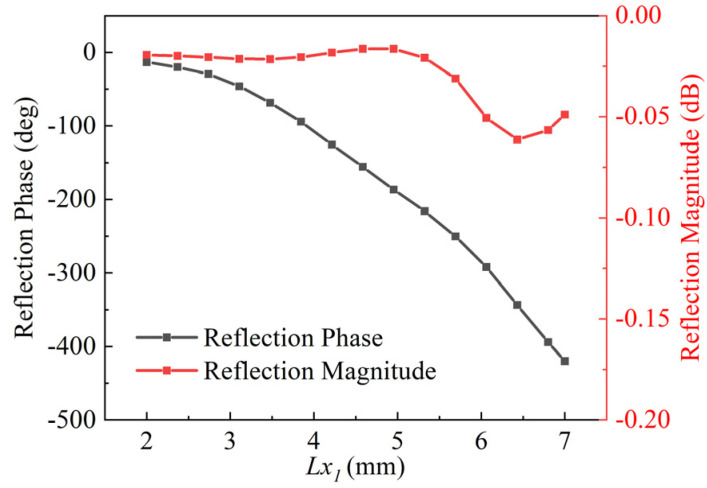
Reflection phase and its amplitude response versus patch size at 16 GHz.

To obtain the reflection coefficient of the designed element, high-frequency simulation software (HFSS) with periodic condition was utilized. A number of parameters have been optimized in order to improve the linearity of the phase shift curve at 16 GHz. These are summarized in Table 1 below. It is concluded that a smooth phase curve with a range over 360° and a maximum dissipation loss is shown to be less than 0.06 dB, as shown in Figure 3.

**Figure 4:**
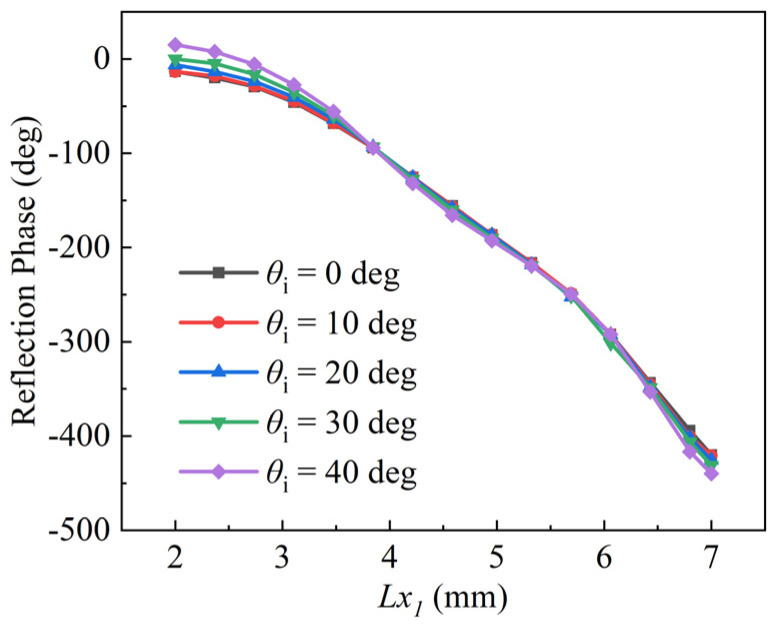
Reflection phase response under different angles of incidence.

For the Σ beam, the radiation performance can mainly be attributed to the center cells of the metasurface, owing to the field strength of the center region being greater than that of the edge region. For the Δ beams, the situation is just the opposite. Different regions will have different incident angles. It is therefore necessary to consider the effects of the incident angles on the reflection phase of the cell. Figure 4 shows phase responses with various incident angles. It can also be observed that the phase curves maintain stability as the incident angle is less than 30°, otherwise phase errors will be introduced. For this reason, the maximum incident angle will be taken into account in the next step of the metasurface design.

## 4. Design of Monopulse Feed and Comparator

**Figure 5:**
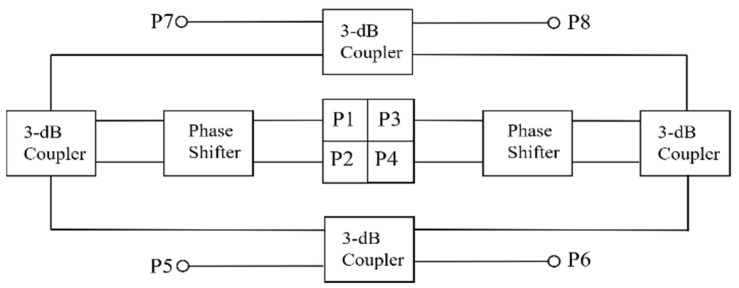
The block diagram of the monopulse feed and comparator.

**Figure 6:**
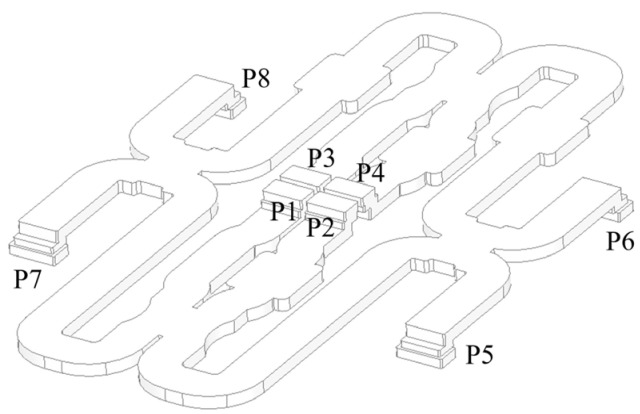
The structure of the monopulse comparator.

**Figure 7:**
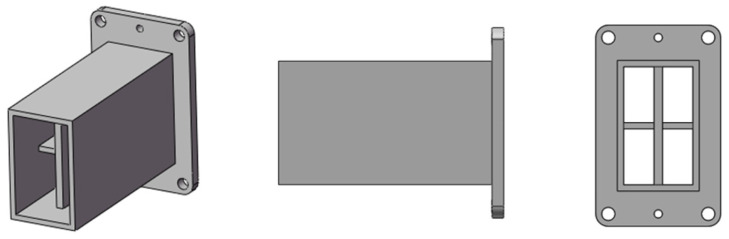
The structure of the monopulse feed.

The monopulse feed and comparator are key components in the tracking radar. In this study, a narrow-wall waveguide is used to design the comparator instead of the SIW which usually has high dielectric loss. The block diagram of the monopulse feed and comparator are presented in Figure 5. There are four 3-dB couplers and four 90° phase shifters in the comparator. For the 3-dB couplers, by adjusting the width of the coupling region, the input power can be divided equally between the through port and the coupling port. Additionally, a 90° phase difference exists between the through port and the coupling port. Instead of adjusting the width or length of the waveguide, the rectangle waveguide to single-ridge waveguide transition is designed for 90° phase shifters, resulting in a shorter phase-shifter section. The whole monopulse comparator is shown in Figure 6, the size is 220 mm × 150 mm × 4 mm, which has low profile. The monopulse comparator has eight ports. The port5 is denoted as Σ port, the port6 and port7 are the Δ ports. The port1–port4 are used to connect with the monopulse feed which adopts a four-horn structure, as shown in Figure 7. Impedance matching between the comparator and feed can be improved by adjusting the height of the partition.

**Figure 8:**
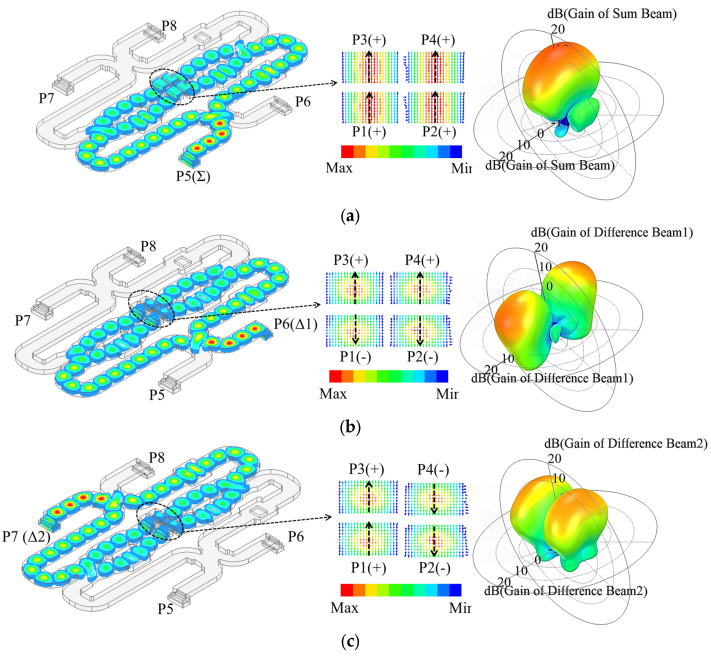
Electric field distributions and radiation diagrams of the monopulse feed and comparator.

Figure 8 illustrates the operation of the monopulse feed and comparator. Figure 8a illustrates the electric field distribution inside the monopulse comparator for Σ port excitation. This distribution enables the monopulse feed to generate a pencil beam with an equi-phase pattern. When exciting the Δ ports, the field distributions are shown in Figure 8b,c, respectively. In this case, the four port fields of the horn are distributed in pairs in reverse phase, which results in a bimodal beam with a pronounced null in boresight. It is possible to obtain a monopulse function through the combination of the sum and difference diagrams.

## 5. Metasurface-Based Main-Reflector and Simulation Results

**Figure 9:**
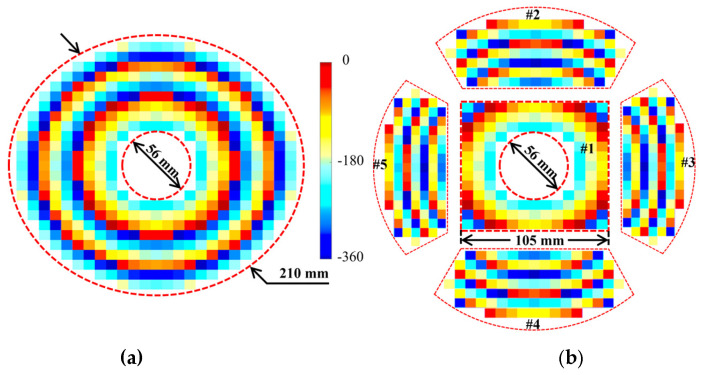
The phase distributions over (**a**) the PMBMR (**b**) the BMBMR.

The cell of the metasurface, the monopulse feed, and comparator have been analyzed and designed in the previous sections. A metasurface-based main reflector will be designed. In the Cassegrain monopulse antenna, the electromagnetic wave is excited by the monopusle feed and comparator is reflected by the sub-reflector, and the main-reflector will transform the secondary radiation wave into well-collimated wave-fronts. For the metasurface-based main-reflector, the desired wave-fronts can be achieved by the specific abrupt-phase discontinuities of the metasurface. In order to obtain the phase distribution of the metasurface, ray tracing is usually employed to analyze the differences of the propagation path. As shown in Figure 1, for the planar metasurface-based main-reflector (PMBMR), path differences are caused by the distance between the virtual focal point *F_1_* and the center position N of an arbitrary element on the metasurface. However, for the bowl-shaped metasurface-based main-reflector (BMBMR), in addition to the above-mentioned path difference, the height difference of the edge cells of the bowl-shaped structure will also cause the path differences. Accordingly, the PMBMR and BMBMR provide phase distributions as follows: $\Phi_{pm}=k(l_{F_{1}N})+\Phi_{0}$ and $\Phi_{bm}=k(l_{F_{1}M}+l_{MM’})+\Phi_{0}$, where k refers to the wave number in free space and Φ_0_ refers a constant value. In accordance with Snell’s generalized law [29], we can obtain the following equations

(1) $$\Phi_{pm}=k\left(\sqrt{x^{2}+y^{2}+F_{m}{}^{2}}-F_{m}\right)+\Phi_{0}$$

(2) $$\Phi_{bm}=k\left(\sqrt{x^{2}+y^{2}+{\left(F_{m}-z\right)}^{2}}+(F_{m}-z)\right)+\Phi_{0}$$

where x, y, and z refer to the coordinate values. The PMBMR and the BMBMR would share the same structure parameters to calculate the phase distributions. The structure parameters are listed as follows: The diameters of the main-reflector and sub-reflector are D_m_ = 210 mm and D_s_ = 60 mm, respectively. A coefficient of eccentricity of five is selected for the sub-reflector, while F_m_ = 80 mm is chosen for the antenna. The distance between the phase center F_2_ and the vertex point O′ of the sub-reflector is fixed at 19.45 mm. The distance between the virtual point F_1_ and the vertex point O′ of the sub-reflector is 12.8 mm. The PMBMR has a maximum incident angle of 53° in this configuration. In the Section 3, the phase errors would be introduced when the incident angle of the cell is larger than 30°; therefore, the folding angle *θ* of the BMBMR is 33°, ensuring that the maximum incident angle is less than 30°, which can minimize the angle-of-incidence effects. Based on these structural parameters, the phase distributions over the PMBMR and the BMBMR are illustrated at 16 GHz in Figure 9. It can be observed that the phase distributions of the PMBMR and BMBMR feature the same halo shape over the first region (#1). In other regions (#2–#5), the phase distributions of the PMBMR maintain the trend but those of the BMBMR become different.

**Figure 10:**
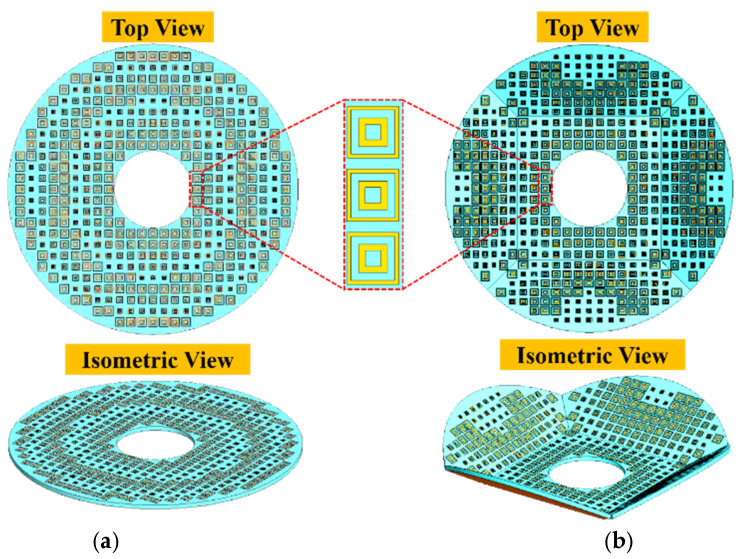
The layouts of (**a**) the PMBMR (**b**) the BMBMR.

**Figure 11:**
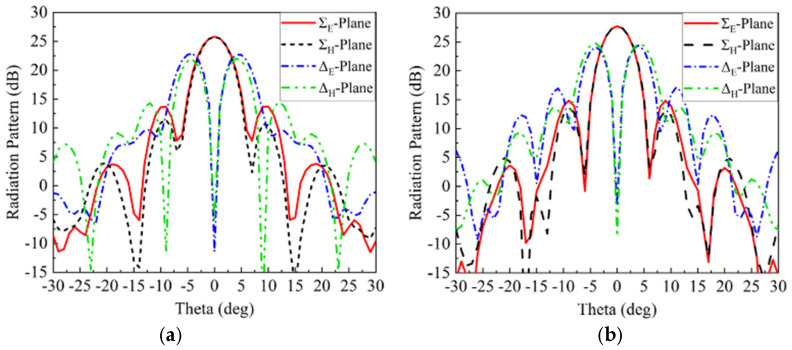
The radiation patterns of the monopulse antenna with (**a**) the PMBMR (**b**) the BMBMR.

The layouts of the PMBMR and BMBMR can be built up based on the relationship between the reflection phase and the size of the cell patch in Figure 3, which is shown in Figure 10. As shown in Figure 1, full wave simulations are performed to verify the designs. The radiation performances of the Σ and Δ beams of the PMBMR and BMBMR are demonstrated in Figure 11. As can be seen, both systems exhibit the typical Σ and Δ patterns. The maximum gains of the Σ beam are 25.6 dB and 27.7 dB, respectively, at 16 GHz with the corresponding aperture efficiencies of 30.7 and 47.5% for the PMBMR and BMBMR. Clearly, the case of BMBMR possesses higher gains and better aperture efficiencies than that of the PMBMR, and this is due to the precise phase compensation of the BMBMR. Moreover, the Δ beams null depth of the both cases are all less than −32 dB, and the maximum gain ratio between the Σ and Δ beams for the PMBMR and BMBMR are 4.6 dB and 4.2 dB, respectively. The gain ratio of the BMBMR is also improved which means the radiation performances of Δ beams are also ameliorated.

## 6. The Fabrication and Measurement Results

**Figure 12:**
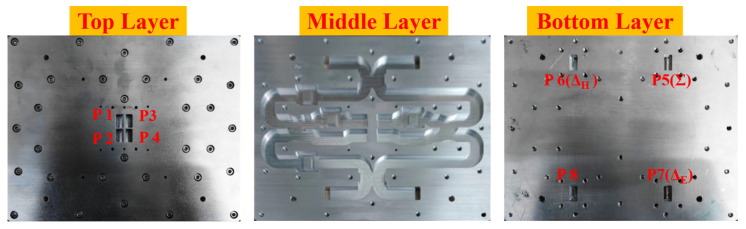
Picture of the manufactured monopulse comparator with the alignment holes before assembling.

**Figure 13:**
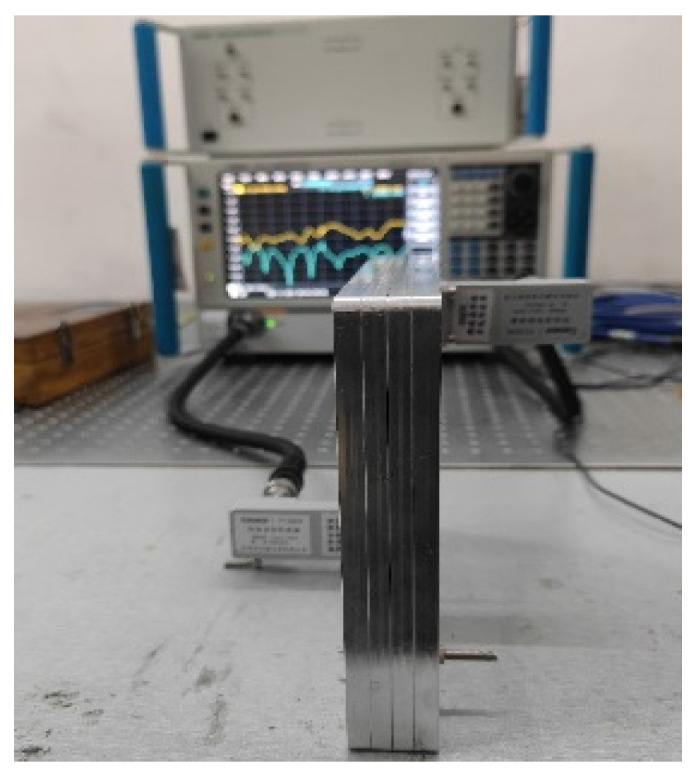
Measurement setup for the fabricated comparator.

Finally, a complete monopulse antenna with BMBMR was fabricated to verify the design procedure and the simulated results; the monopulse comparator is fabricated by using CNC milling machining in aluminum. Figure 12 shows the fabricated comparator. The overall size of the comparator is 220 mm × 150 mm × 14.4 mm. The fabricated prototype has a simple mechanical assembly and the metallic layers are simply held in their respective positions by using several guiding pins and screws. The scattering parameters have been measured by connecting two identical comparators back-to-back, as shown in Figure 13. We have used a vector network analyzer (VNA) to perform the measurements. There are eight ports on the comparator: the ports 5–8 are the input ports which place on the bottom layer, and the ports 1–4 are the output ports which locate at the top layer. Two-port S-parameter measurement was performed between each input port of the one comparator and that of the other comparator one at a time.

**Figure 14:**
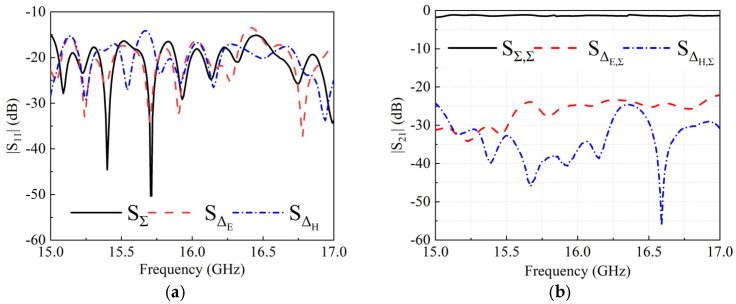
(**a**) Measured reflection coefficients and (**b**) measured transmission coefficients of the designed comparator.

Figure 14a illustrates the measured reflection coefficients for the Σ and Δ ports. For the frequency band 15–17 GHz, all $\left|S_{\sum }\right|$, $\left|S_{\Delta_{E}}\right|$, $\left|S_{\Delta_{H}}\right|$ are below −12 dB(VSWR = 1.7:1). The measured transmission coefficients from the Σ port to the opposite ports are shown in Figure 14b. There is a transmission loss of 1.4 dB over the frequency band and a port isolation of less than −22 dB.

**Figure 15:**
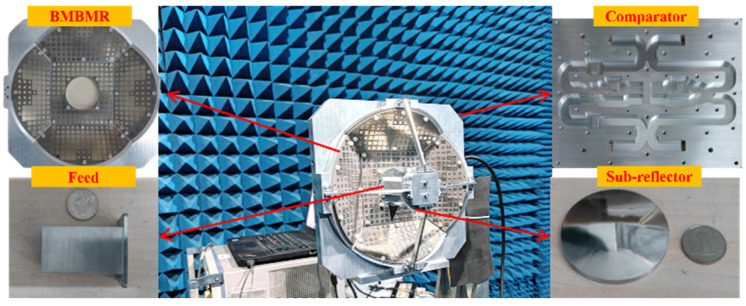
Measurement setup for the fabricated metasurface-based monopulse antenna.

Figure 15 illustrates the assembly of the metasurface-based monopulse antenna. The manufacturing process for the feed and sub-reflector prototype is the same as the comparator in Figure 12. The metasurfaces are fabricated by means of PCBs. We measure the far-field radiation characteristics of the antenna in an anechoic chamber.

**Figure 16:**
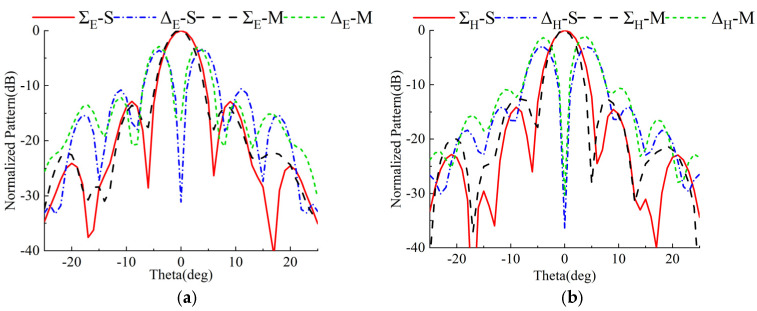
Simulated and measured normalized sum and difference radiation patterns at 16 GHz. (**a**) E-plane and (**b**) H-plane.

A comparison of measured and simulated radiation patterns at 16 GHz at the boresight can be seen in Figure 16. Measured and simulated results are represented by symbols M and S, respectively. Compared to simulated patterns, the measured patterns are in good agreement. The measured gain of the Σ beam is 27.1 dB by eliminating the loss of the designed comparator, which the aperture efficiency is about 41.4%. As can be seen, a 0.6 dB loss is observed between the measured and simulated gains. Prototypes fabricated with errors in measurement and alignment cause this. In addition, in both planes, the first sidelobe level is less than 12 dB, the 3 dB beam width is 5.1°, and the Δ beams null depth are below −25 dB. The gain ratios between the Σ and Δ beams are 2.8 dB and 3.7 dB, respectively.

A comparison of the performance of the monopulse antennas is shown in Table 2. According to this table, the proposed antenna system also has fair characteristics in terms of the gain, the aperture efficiency, and the gain ratio of the Σ and Δ patterns.

## 7. Conclusions

A novel metasurface-based monopulse Cassegrain antenna is proposed in this paper. The metasurface comprised the concentric square rings that are fixed on a bowl-shaped main-reflector for minimizing the angle-of-incidence effects. For the sake of a sufficient sampling rate, the sub-reflector adopts the metal hyperboloid structure instead of the metasurface. In order to reduce the transmission loss, a narrow-wall waveguide monopulse comparator, which includes 3-dB couplers and the optimized waveguide phase shifters, has been utilized to construct the monopulse comparator. The prototype of the proposed antenna is designed, fabricated, and measured at Ku-band. The measured transmission loss of the monopulse comparator is about 1.4 dB over the frequency band 15–17 GHz, which has low loss. The measured results of the complete antenna show that the gain ratios between the Σ and Δ beams are 2.8 dB and 3.7 dB in both planes, respectively. The Δ beams null depths are also below −25 dB in both planes. More importantly, the peak gain of the Σ beam at 16 GHz is 27.1 dB, and the corresponding aperture efficiency is about 41.4%. This indicates the proposed structure is beneficial for improving the radiation performance of the Σ and Δ beams, which is suitable for tracking systems.

## Figures and Tables

**Table 1:** Optimized Parameters of The Proposed Cell.

P	k	h	w_1_	w_2_
8 mm	0.65	3.175 mm	0.5 mm	1 mm

**Table 2:** Performance Comparison with Other Structures.

Ref.	Freq (GHz)	Maximum Gain of Σ Pattern (dBi)	Aperture Size	Measured Aperture Efficiency	Gain Ratio Σ/Δ Patterns (dB)
[4]	35	35.6	140 mm, circular	19.1%	3.5
[5]	5.83	12.9	109 mm × 174 mm	21%	>3
[25]	10	21.5	200 mm × 200 mm	32.2%	4.7/7.56
[26]	35	29.4	170 mm, circular	22.43%	3/5
This work	16	27.1	210 mm, circular	41.4%	2.8/3.7

## Data Availability

The data supporting the findings of this study can be made available to the genuine readers after contacting the corresponding authors.
